# Supplementary material for: Conformational trajectory of the HIV-1 fusion peptide during CD4-induced envelope opening
Source: Nat Commun. 2025 May 17;16:4595. doi: 10.1038/s41467-025-59721-2 (PMC12085566; doi:10.1038/s41467-025-59721-2)
Supplement: Supplementary file 1 — Supplementary Information [file 41467_2025_59721_MOESM1_ESM.pdf]

Supplementary Table 1. Cryo-EM data collection and refinement statistics.

	BG505 SOSIP.664 bound to 3-sCD4, 3-17b Fab and 3-VRC34.01 Fab	BG505 SOSIP.664 bound to 3-sCD4, 3-17b Fab and 2-VRC34.01 Fab	BG505 SOSIP.664 bound to 3-sCD4, 3-17b Fab and 1-VRC34.01 Fab	BG505 SOSIP.664 bound to 3-sCD4, 3-VRC34.01 Fab with one gp120 rotated	BG505 SOSIP.664 bound to 3-sCD4, 3-VRC34.01 Fab with two gp120 rotated
	**Population 1**	**Population 2**	**Population 3**	**Population 4**	**Population 5**
**PDB ID**	9D90			9D8Y	9D98
**EMDB ID**	EMD-46655	EMD-46671	EMD-46672	EMD-46653	EMD-46670
**Data Collection and processing**					
Microscope	FEI Titan Krios				
Detector	Gatan K3				
Magnification	81000				
Voltage (kV)	300				
Electron exposure (e-/Å^2)	58.7	58		59.1	
Defocus Range (μm)		2.4 to 0.8			
Pixel size (Å)	1.08				
Micrographs collected	18,253	21,125		23,157	
Reconstruction software	cryoSPARC				
Symmetry imposed	C1	C1	C1	C1	C1
Initial particle images (no.)	1,473,619	1,105,644	1,105,644	998,476	
Final particle images (no.)	1,028,498	58,240	25,613	274,345	138,204
Map resolution (Å)	3.9	4.9	6.4	4.1	4.2
FSC threshold	0.143	0.143	0.143	0.143	0.143
**Model composition**					
Nonhydrogen atoms	24524			18800	19100
Protein residues	3151			2384	2441
**R.M.S. deviations**					
Bond lengths (Å)	0.005			0.005	0.005
Bond angles (°)	0.964			0.944	1.04
**Validation**					
MolProbity score	1.64			1.62	1.68
Clashscore	4.78			4.25	4.98
Favored rotamers (%)	99.94			99.92	99.75
**Ramachandran plot**					
Favored regions (%)	94.15			93.76	93.74
Disallowed regions (%)	0.06			0.09	0.25

Supplementary Table 2. Summary of structural states identified in this study.

	Population 4	Population 5	Population 1	Population 2	Population 3
Ligands	CD4, VRC34.01	CD4, VRC34.01	CD4, 17b, VRC34.01	CD4, 17b, VRC34.01	CD4, 17b, VRC34.01
VRC34.01 bound	✓✓✓	✓✓✓	✓✓✓	✓✓X	✓XX
Bridging sheet formed	✓XX	✓✓X	✓✓✓	✓✓✓	✓✓✓
α0 helix formed	✓XX	✓✓X	✓✓✓	✓✓✓	✓✓✓
Fusion peptide accessible for antibody binding	✓✓✓	✓✓✓	✓✓✓	✓✓X	✓XX
Extent of receptor-induced Env conformational change*					

✓/X indicates structural state per protomer

* Shaded by extent of Env opening, light red indicating less open Env and dark red indicating more open Env.

Supplementary Table 3. List of structures used for vector analysis in Figure 2E.

PDB ID	Ligands	Env name
9D90 (Population 1, P1)	sCD4, 17b Fab, VRC34.01 Fab	BG505 SOSIP.664
5VN3	sCD4, 17b Fab	B41 SOSIP.664
5VN8	b12 Fab	B41 SOSIP.664
6CM3	sCD4, 17b Fab, 8ANC195 Fab	BG505 SOSIP.664
6EDU	sCD4, 21C Fab, 8ANC195 Fab	B41 SOSIP.664
6V8Z	VRC03 Fab, 10-1074 Fab	BG505 SOSIP.664-F14/Vt8
4TVP	PGT122 Fab, 35O22 Fab	BG505 SOSIP.664
4ZMJ	None	BG505 SOSIP.664
5ACO	PGT128 Fab	BG505 SOSIP.664
5C7K	PGT128 Fab, 8ANC195 Fab	BG505 SOSIP.664
5CEZ	Early putative precursor of the PGT121 family	BG505 SOSIP.664
5CJX	8ANC195 Fab	BG505 SOSIP.664
5D9Q	PGT122 Fab, scFv NIH45-46	BG505 SOSIP.664
5FUU	PGT151 Fab	JR-FL EnvdCT
5FYJ	PGT122 Fab, 35O22 Fab, VRC01 Fab	HIV-1 Clade G X1193.c1 SOSIP.664
5FYK	PGT122 Fab, 35O22 Fab, VRC01 Fab	JR-FL SOSIP.664
5FYL	PGT122 Fab, 35O22 Fab	BG505 SOSIP.664
5I8H	PGT122 Fab, VRC34.01 Fab	BG505 SOSIP.664
5T3X	IOMA, 10-1074.	BG505 SOSIP.664
5T3Z	IOMA, 10-1074.	BG505 SOSIP.664
5U1F	sCD4, PGT145 Fab	BG505 DS-SOSIP
5U7M	BMS-378806, PGT122 Fab, 35O22 Fab	BG505 SOSIP.664
5U7O	BMS-626529, PGT122 Fab, 35O22 Fab	BG505 SOSIP.664
5UM8	35022 Fab,PGT124 Fab	16055 NFL TD CC (T569G)
5UTF	PGT122 Fab, 35O22 Fab	DS-SOSIP.6mut BG505 gp140 HIV-1
5UTY	PGT122 Fab, 35O22 Fab	DS-SOSIP.mut4 BG505 gp140 HIV-1
5V7J	3H+109L Fab, 35O22 Fab	BG505 SOSIP.664
5V8L	3BNC117 Fab, PGT145 Fab	BG505 SOSIP.664
5V8M	3BNC117 Fab	BG505 SOSIP.664
5VIY	BG1 Fab, 8ANC195 Fab	BG505 SOSIP.664
5VJ6	PG9 Fab, 8ANC195 Fab	BG505 SOSIP.664
5WDU	PGT122 Fab, 35O22 Fab, NIH45-46 scFv	BG505 SOSIP.664 H72C-H564C
6B0N	PGT122 Fab, PGV19 Fab	BG505 isolate (NFL construct)
6CCB	10-1074 Fab	253-11 SOSIP trimer
6CDE	VRC03 Fab, PGT122 Fab, vFP20.01 Fab	BG505 DS-SOSIP
6CDI	VRC03 Fab, PGT122 Fab, vFP16.02 Fab	BG505 DS-SOSIP
6CE0	PGT124 Fab, 35O22 Fab	H078.14 UFO-BG
6CH7	BG18 Fab, 35O22 Fab	BG505 SOSIP.664
6CH8	BG18 Fab, 35O22 Fab	BG505 SOSIP.664
6CH9	BG18 Fab, 35O22 Fab	B41 SOSIP.664
6CHB	BG18 Fab, IOMA Fab	BG505 SOSIP.664
6CK9	3H109L Fab, 35O22 Fab	HIV-1 ConC_Base0
6CUE	VRC03 Fab, PGT122 Fab, vFP7.04 Fab	BG505 DS-SOSIP
6CUF	VRC03 Fab, PGT122 Fab, vFP1.01 Fab	BG505 DS-SOSIP
6DE7	PGT122 Fab, 35O22 Fab	BG505 SOSIP.664
6DID	Fabs from immunized rabbit #3417 post-boost#1	BG505 SOSIP.664
6E5P	2G12 Fab, VRC03 Fab	BG505 DS-SOSIP
6IEQ	PGT124 Fab, 35O22 Fab	HIV-1 Env ConM SOSIP.v7
6-Mar	PGT151 Fab	BG505 delCT N332T
6MCO	PGT124 Fab, 35O22 Fab	B41 SOSIP.664
6MDT	PGT124 Fab, 35O22 Fab	B41 SOSIP.664
6MN7	BF520.1 antigen binding fragment	BG505 SOSIP.664
6MPG	PGT122 Fab, VRC03 Fab, A12V163-b.01 Fab	BG505 DS-SOSIP
6MPH	PGT122 Fab, VRC03 Fab, DF1W-a.01 Fab	BG505 DS-SOSIP
6MTJ	BMS-378806, 3H109L Fab, 35O22 Fab	BG505 SOSIP.664
6MTN	Compound 484, 3H109L Fab, 35O22 Fab	BG505 SOSIP.664
6MU6	BMS-814508, 3H109L Fab, 35O22 Fab	BG505 SOSIP.664
6MU7	BMS-818251, 3H109L Fab, 35O22 Fab	BG505 SOSIP.664
6MU8	BMS-386150, 3H109L Fab, 35O22 Fab	BG505 SOSIP.664
6MUF	3H109L Fab, 35O22 Fab	B41 SOSIP.664
6MUG	BMS-386150, 3H109L Fab, 35O22 Fab	B41 SOSIP.664
6N1V	A12V163-a.01 Fab, VRC03 Fab, PGT122 Fab	BG505 DS-SOSIP
6N1W	DFPH-a.15 Fab, VRC03 Fab, PGT122 Fab	BG505 DS-SOSIP
6NC2	ACS202 Fab	AMC011 v4.2 SOSIP Env
6NC3	VRC34 Fab	AMC011 v4.2 SOSIP Env
6NF2	0PV-c.01 Fab, VRC03 Fab, PGT122 Fab	BG505 DS-SOSIP
6NIJ	PGT145 Fab	AMC011 HIV-1 Env
6NM6	N6 FR3-03 scFv, 3H109L Fab, 35O22 scFv	BG505 SOSIP.664
6NNF	FR3-03 scFv, 3H109L Fab, 35O22 scFv	BG505 SOSIP.664
6NNJ	CH31 scFv, 3H109L Fab, 35O22 scFv	BG505 SOSIP.664
6OHY	None	Chimpanzee SIV Env trimeric ectodomain
6OKP	SF12 Fab, 10-1074 Fab	B41 SOSIP.664
6OLP	PGT151 Fab	HIV-1 Env AMC011
6ORN	10-1074 Fab	BG505 SOSIP.664 based immunogen RC1
6ORO	Ab874NHP Fab	BG505 SOSIP.664 based immunogen RC1
6ORP	Ab897NHP Fab	BG505 SOSIP.664 based immunogen RC1
6ORQ	Ab275NHP Fab	BG505 SOSIP.664 based immunogen RC1
6OSY	VRC03 Fab, PGT122 Fab, 0PV-a.01 Fab	BG505 DS-SOSIP
6OT1	VRC03 Fab, PGT122 Fab, 0PV-b.01 Fab	BG505 DS-SOSIP
6UDA	CH235UCA Fab	CH505.N279K.G458Y.SOSIP.664

Supplementary Table 4. Relative state occupancy and fitting parameters of conformational distributions of the virus Env observed from two different structural perspectives, with reference to Figure 4E.

**BG505 Virus Env**	Curve fitting R^2	RMSE	Pre-triggered	Pre-fusion closed	Three-CD4 open
			μ: 0.1 ± 0.02	μ: 0.60 ± 0.03	μ: 0.3 ± 0.03
			σ: 0.07 ± 0.01	σ: 0.15 ± 0.02	σ: 0.1 ± 0.01
**$N136_{TAG}$ $S401_{TAG}$**					
Ligand-free	0.9891	9.4074e-04	52% ± 07%	18% ± 12%	30% ± 10%
+ VRC34	0.9669	0.0012	35% ± 11%	45% ± 14%	20% ± 11%
+ sCD4 + 17b + VRC34	0.9891	7.044e-04	28% ± 06%	38% ± 14%	34% ± 14%
+ sCD4 + 17b	0.9791	9.2681e-04	26% ± 09%	33% ± 14%	41% ± 13%

**BG505 Virus Env**	Curve fitting R^2	RMSE			
			μ: 0.55 ± 0.05	μ: 0.28 ± 0.03	μ: 0.10 ± 0.02
			σ: 0.15 ± 0.02	σ: 0.1 ± 0.01	σ: 0.07 ± 0.01
**V4-A1 $R542_{TAG}$**					
Ligand-free	0.9914	5.0798e-04	53% ± 07%	30% ± 07%	17% ± 03%
+ VRC34	0.9903	7.6231e-04	36% ± 16%	47% ± 12%	17% ± 13%
+ sCD4 + 17b + VRC34	0.9946	4.9572e-04	33% ± 06%	33% ± 11%	34% ± 07%
+ sCD4 + 17b	0.9914	7.3402e-04	32% ± 10%	27% ± 14%	41% ± 10%

A pair of fluorophores labeled on different Env sites result in different FRET signals. In each FRET structural perspective, FRET histograms were fitted into the sum of three distinct Gaussian distributions with defined means (μ) and deviations (σ) of the individual Gaussian/normal distributions. μ and σ were determined based on the observation of unbiased raw FRET signals and were further determined using hidden Markov modeling. As a measure of uncertainty, we presented the probability of each state Env occupies as mean + s.e.m. R^2 and RMSE (Root Mean Square Deviation) evaluate the goodness of fitting.

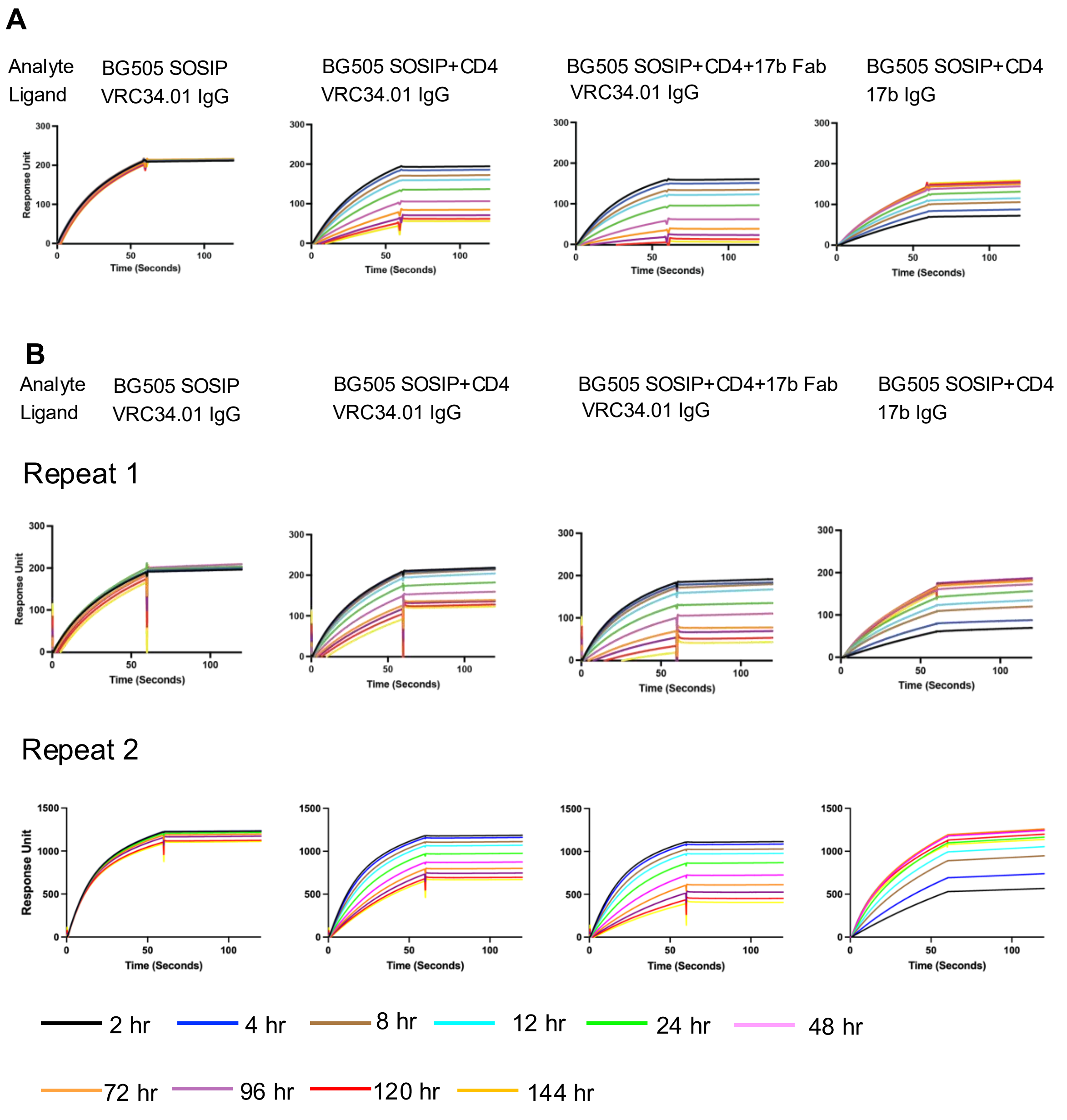

Supplementary Figure 1. CD4-induced conformational changes in HIV-1 Env measured using SPR. Blank subtracted, double referenced sensorgrams obtained by injecting on a ligand IgG surface the analytes at specific time-points post-incubation. **A)** Incubations and measurements performed at 25 °C. **B)** Incubations and measurements performed at 37 °C. Data shown are representative of at least two independent experiments. Two independent repeats are shown in panel B, performed at two levels of immobilization. Results were equivalent, showing progressive increase in 17b binding and reduction of VRC34.01 binding post CD4 addition to Env (an effect that was enhanced when 17b Fab was added together with CD4). Source data are provided as Source Data files.

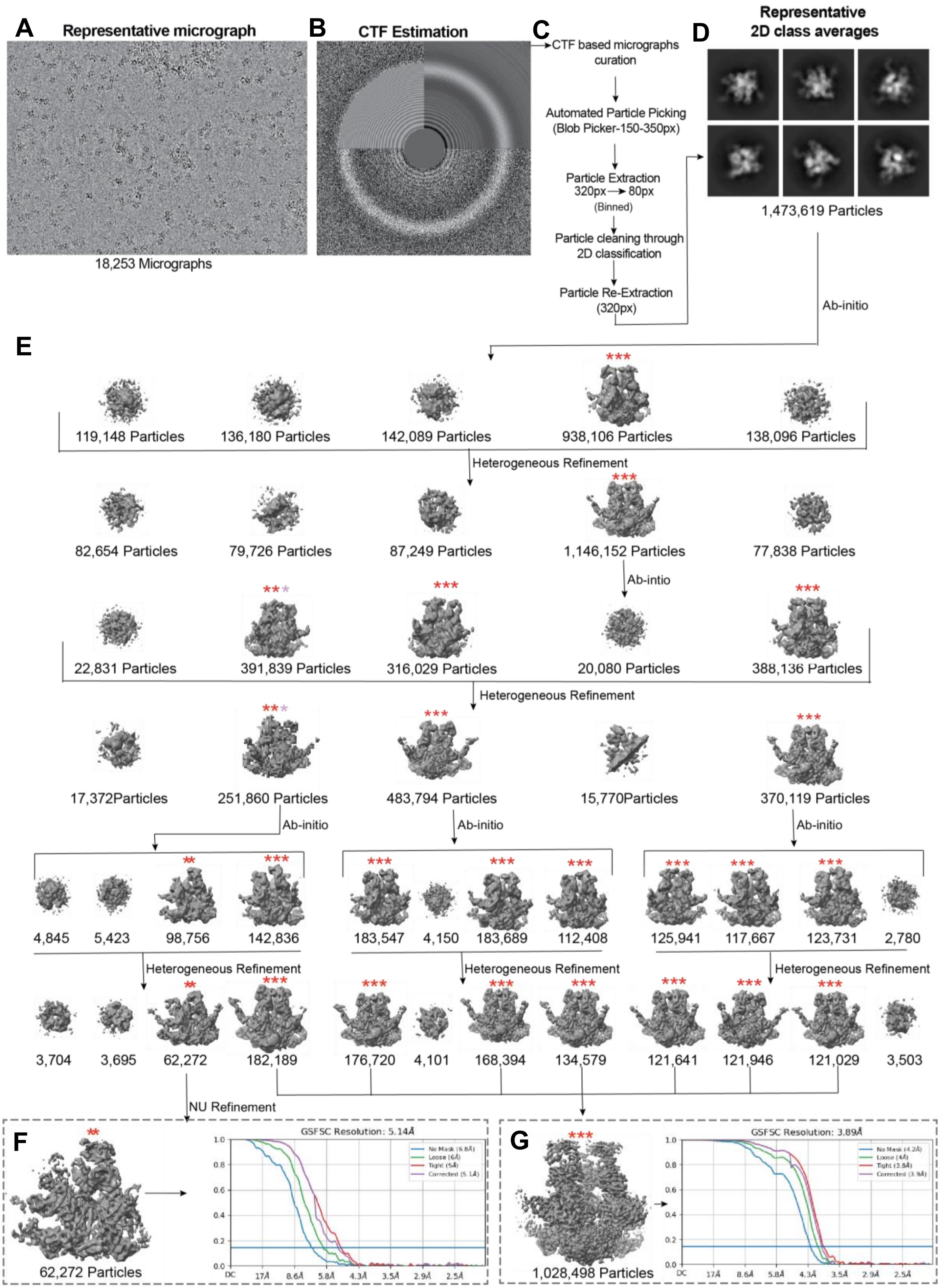

A
Representative micrograph
18,253 Micrographs
B
CTF Estimation
C
CTF based micrographs curation
Automated Particle Picking (Blob Picker-150-350px)
Particle Extraction 320px → 80px (Binned)
Particle cleaning through 2D classification
Particle Re-Extraction (320px)
D
Representative 2D class averages
1,473,619 Particles
Ab-initio
E
119,148 Particles
136,180 Particles
142,089 Particles
938,106 Particles
138,096 Particles
Heterogeneous Refinement
82,654 Particles
79,726 Particles
87,249 Particles
1,146,152 Particles
77,838 Particles
Ab-intio
22,831 Particles
391,839 Particles
316,029 Particles
20,080 Particles
388,136 Particles
Heterogeneous Refinement
17,372Particles
251,860 Particles
483,794 Particles
15,770Particles
370,119 Particles
Ab-initio
Ab-initio
Ab-initio
4,845
5,423
98,756
142,836
183,547
4,150
183,689
112,408
125,941
117,667
123,731
2,780
Heterogeneous Refinement
Heterogeneous Refinement
Heterogeneous Refinement
3,704
3,695
62,272
182,189
176,720
4,101
168,394
134,579
121,641
121,946
121,029
3,503
NU Refinement
F
62,272 Particles
GSFSC Resolution: 5.14Å
No Mask (6.8Å)
Loose (6Å)
Tight (5Å)
Corrected (5.1Å)
DC
17Å
8.6Å
5.8Å
4.3Å
3.5Å
2.9Å
2.5Å
G
1,028,498 Particles
GSFSC Resolution: 3.89Å
No Mask (4.2Å)
Loose (4Å)
Tight (3.8Å)
Corrected (3.9Å)

Supplementary Figure 2. Cryo-EM data processing for BG505 SOSIP.664 incubated with CD4 and 17b for 1.3 hours at 25 °C followed by incubation with VRC34.01 Fab for 30 minutes prior to vitrification. **A)** Representative frame aligned micrograph. **B)** Representative contrast transfer function (CTF) fit.. **C)** Data Processing workflow, leading to, **D)** Representative 2D class averages from 2D classification, followed by **,** **E)** 3D classifications and refinements. **F)** Refined map of Env bound to 3 molecules of CD4, 3 molecules of 17b Fabs, and 2 molecule of VRC34.01 Fabs (indicated by 2 red asterisks). **G)** Refined map of Env bound to 3 molecules of CD4, 3 molecules of 17b Fabs, and 3 molecule of VRC34.01 Fabs (indicated by 3 red asterisks). Fourier shell correlation (FSC) curves for refined 3D maps shown in panels **F** and **G** with the horizontal blue line showing FSC threshold value of 0.143.

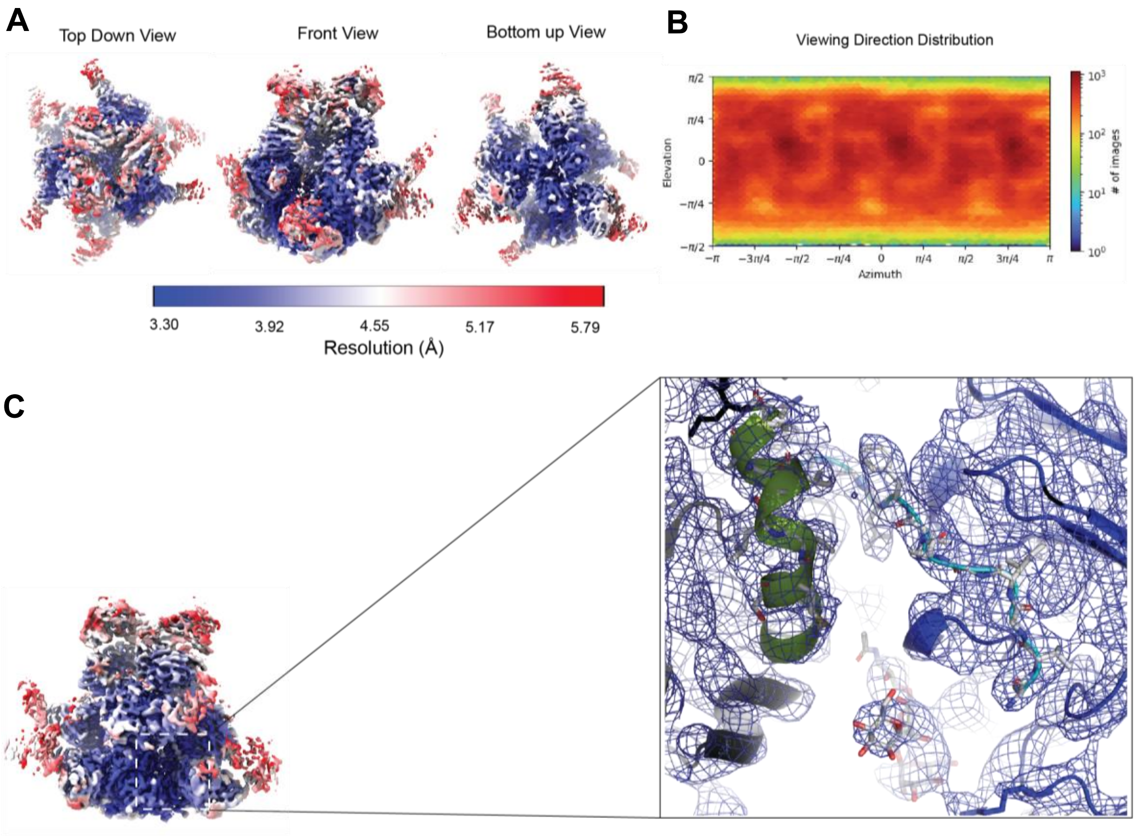

Supplementary Figure 3. Particle distribution and local resolution estimation for cryo-EM dataset of BG505 SOSIP.664 incubated with CD4 and 17b for 1.3 hours followed by incubation with VRC34.01 Fab for 30 minutes prior to vitrification. **A)** Refined cryo-EM map of Env complex with CD4 and 17b, bound to 3 VRC34.01 Fabs, colored by local resolution. **B)** Particle distribution. **C)** Zoomed in view of region around the FP showing the map as a blue mesh (cryo-EM map contoured at a level of 0.143 in ChimeraX) with underlying fitted model shown in cartoon representation and glycans shown as sticks. The FP is shown both in cartoon representation (colored cyan) and as light grey sticks colored by atom.

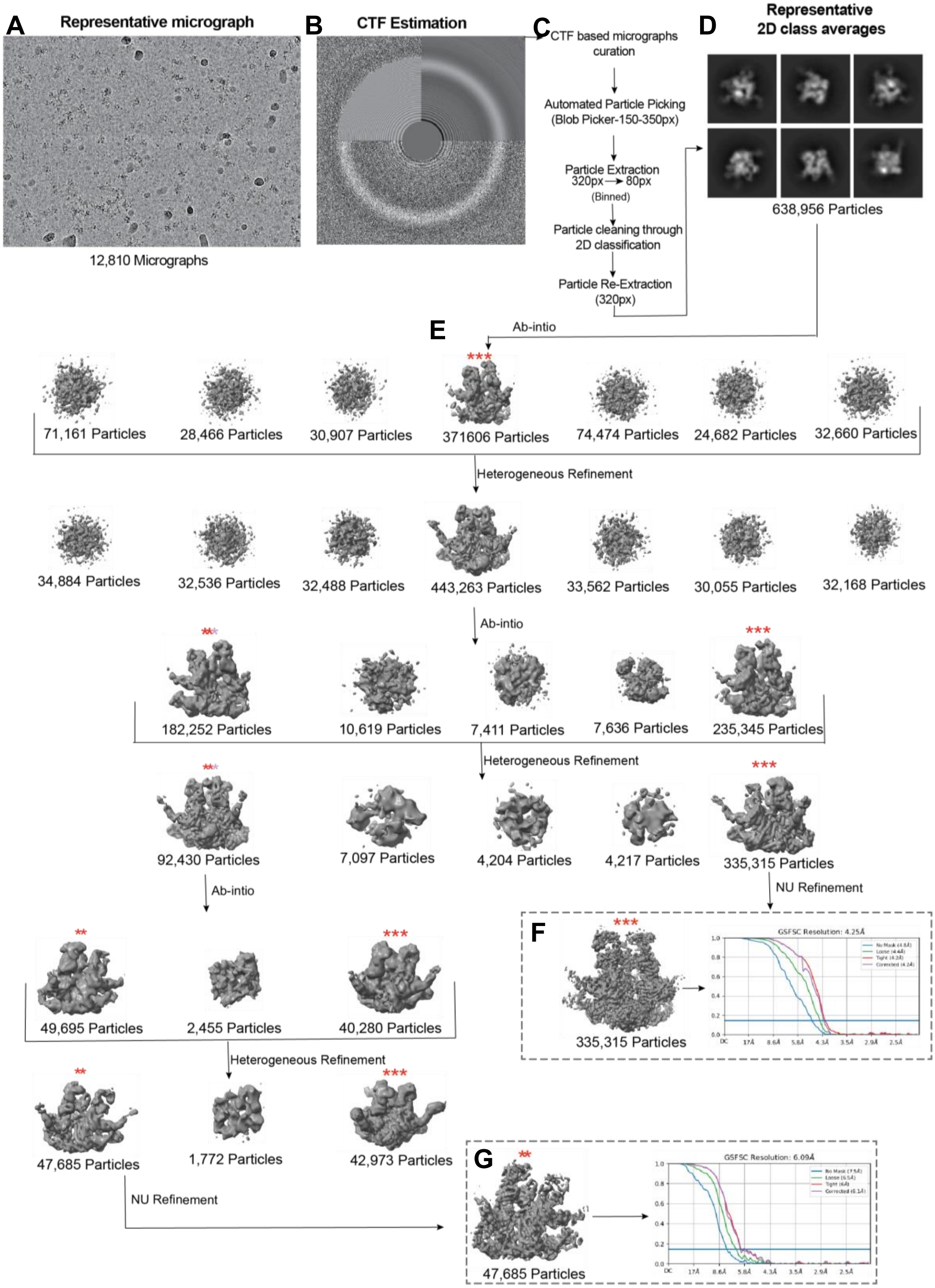

A Representative micrograph
12,810 Micrographs
B CTF Estimation
C CTF based micrographs curation
Automated Particle Picking (Blob Picker-150-350px)
Particle Extraction 320px → 80px (Binned)
Particle cleaning through 2D classification
Particle Re-Extraction (320px)
D Representative 2D class averages
638,956 Particles
E Ab-intio
71,161 Particles
28,466 Particles
30,907 Particles
371606 Particles
74,474 Particles
24,682 Particles
32,660 Particles
Heterogeneous Refinement
34,884 Particles
32,536 Particles
32,488 Particles
443,263 Particles
33,562 Particles
30,055 Particles
32,168 Particles
Ab-intio
182,252 Particles
10,619 Particles
7,411 Particles
7,636 Particles
235,345 Particles
Heterogeneous Refinement
92,430 Particles
7,097 Particles
4,204 Particles
4,217 Particles
335,315 Particles
Ab-intio
NU Refinement
49,695 Particles
2,455 Particles
40,280 Particles
F
335,315 Particles
GSFSC Resolution: 4.25Å
Heterogeneous Refinement
47,685 Particles
1,772 Particles
42,973 Particles
NU Refinement
G
47,685 Particles
GSFSC Resolution: 6.09Å

Supplementary Figure 4. Cryo-EM data processing for BG505 SOSIP.664 incubated with CD4 and 17b for 20 hours at 25 °C followed by incubation with VRC34.01 Fab for 30 minutes prior to vitrification. **A)** Representative frame aligned micrograph. **B)** Representative contrast transfer function (CTF) fit. **C)** Data Processing workflow, leading to, **D)** Representative 2D class averages from 2D classification, followed by, **E)** 3D classifications and refinements. **F)** Refined map of Env bound to 3 molecules of CD4, 3 molecules of 17b Fabs, and 3 molecule of VRC34.01 Fabs (indicated by 3 red asterisks. **G)** Refined map of Env bound to 3 molecules of CD4, 3 molecules of 17b Fabs, and 2 molecule of VRC34.01 Fabs (indicated by 2 red asterisks). Fourier shell correlation (FSC) curves for refined 3D maps shown in panels **F** and **G** with the horizontal blue line showing FSC threshold value of 0.143.

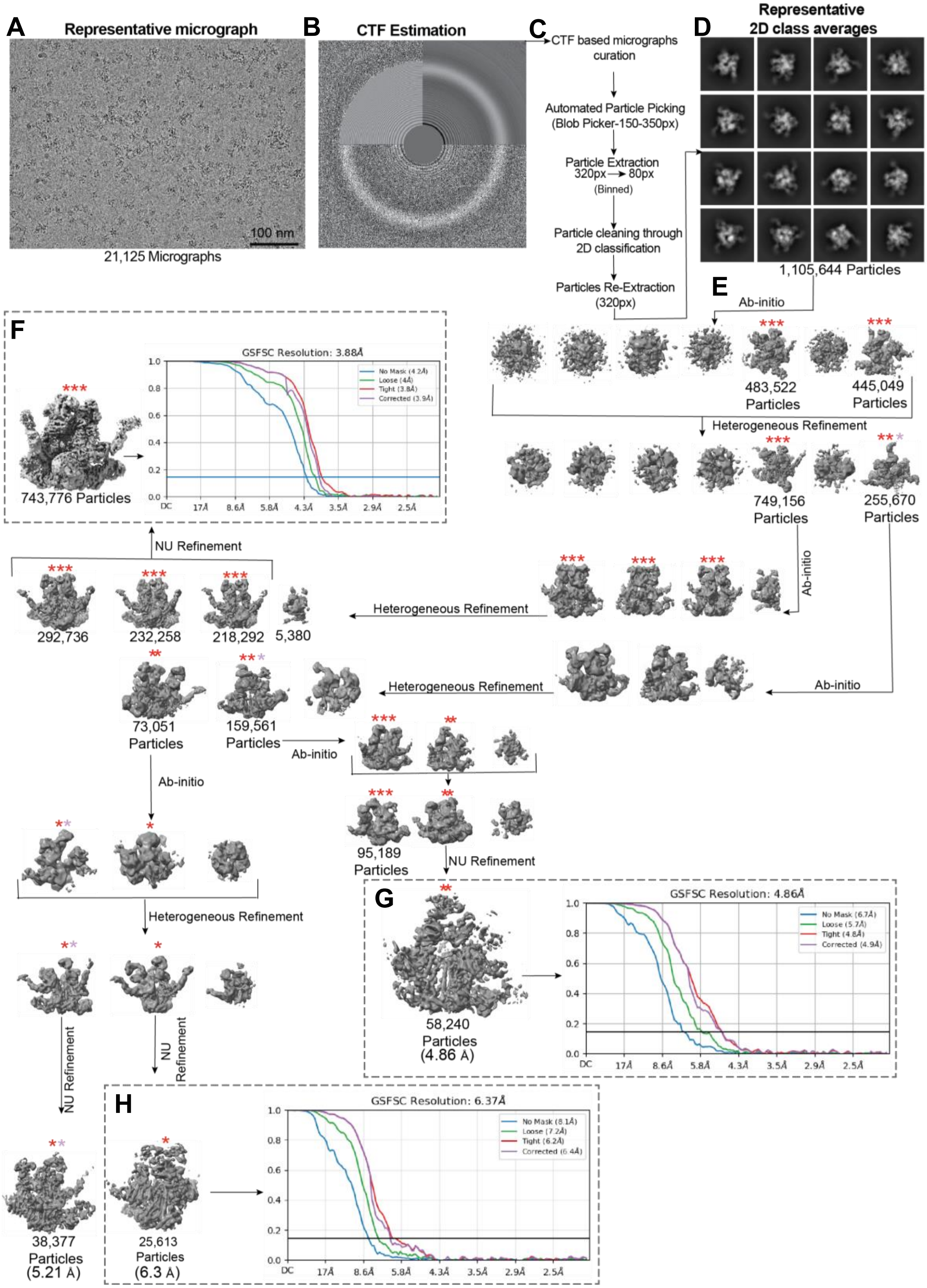

A
Representative micrograph
100 nm
21,125 Micrographs
B
CTF Estimation
C
CTF based micrographs curation
Automated Particle Picking (Blob Picker-150-350px)
Particle Extraction 320px → 80px (Binned)
Particle cleaning through 2D classification
Particles Re-Extraction (320px)
D
Representative 2D class averages
1,105,644 Particles
E
Ab-initio
483,522 Particles
445,049 Particles
Heterogeneous Refinement
749,156 Particles
255,670 Particles
Ab-initio
Heterogeneous Refinement
Ab-initio
Heterogeneous Refinement
F
GSFSC Resolution: 3.88Å
No Mask (4.2Å)
Loose (4Å)
Tight (3.8Å)
Corrected (3.9Å)
743,776 Particles
NU Refinement
292,736
232,258
218,292
5,380
73,051 Particles
159,561 Particles
Ab-initio
Ab-initio
95,189 Particles
NU Refinement
G
GSFSC Resolution: 4.86Å
No Mask (6.7Å)
Loose (5.7Å)
Tight (4.8Å)
Corrected (4.9Å)
58,240 Particles (4.86 Å)
Heterogeneous Refinement
NU Refinement
NU Refinement
H
GSFSC Resolution: 6.37Å
No Mask (8.1Å)
Loose (7.2Å)
Tight (6.2Å)
Corrected (6.4Å)
38,377 Particles (5.21 Å)
25,613 Particles (6.3 Å)

Supplementary Figure 5. Cryo-EM data processing for BG505 SOSIP.664 incubated with CD4 and 17b for 3 days at 25 °C followed by incubation with VRC34.01 Fab for 30 minutes prior to vitrification. **A)** Representative frame aligned micrograph. **B)** Representative contrast transfer function (CTF) fit. **C)** Data Processing workflow, leading to, **D)** Representative 2D class averages from 2D classification, followed by , **E)** 3D classifications and refinements. **F)** Refined map of Env bound to 3 molecules of CD4, 3 molecules of 17b Fabs, and 3 molecule of VRC34.01 Fabs (indicated by 3 red asterisks). **G)** Refined map of Env bound to 3 molecules of CD4, 3 molecules of 17b Fabs, and 2 molecule of VRC34.01 Fabs (indicated by 2 red asterisks). **H)** Refined map of Env bound to 3 molecules of CD4, 3 molecules of 17b Fabs, and 1 molecule of VRC34.01 Fab. (indicated by 1 red asterisk). Fourier shell correlation (FSC) curves for refined 3D maps shown in panels **F-H** with the horizontal blue line showing FSC threshold value of 0.143. The asterisk indicate the number of VRC34.01 bound in each cryo-EM map. The red asterisks indicate well-defined density for the VRC34.01 Fab. The pink asterisks indicate partial density for the VRC34.01 Fab.

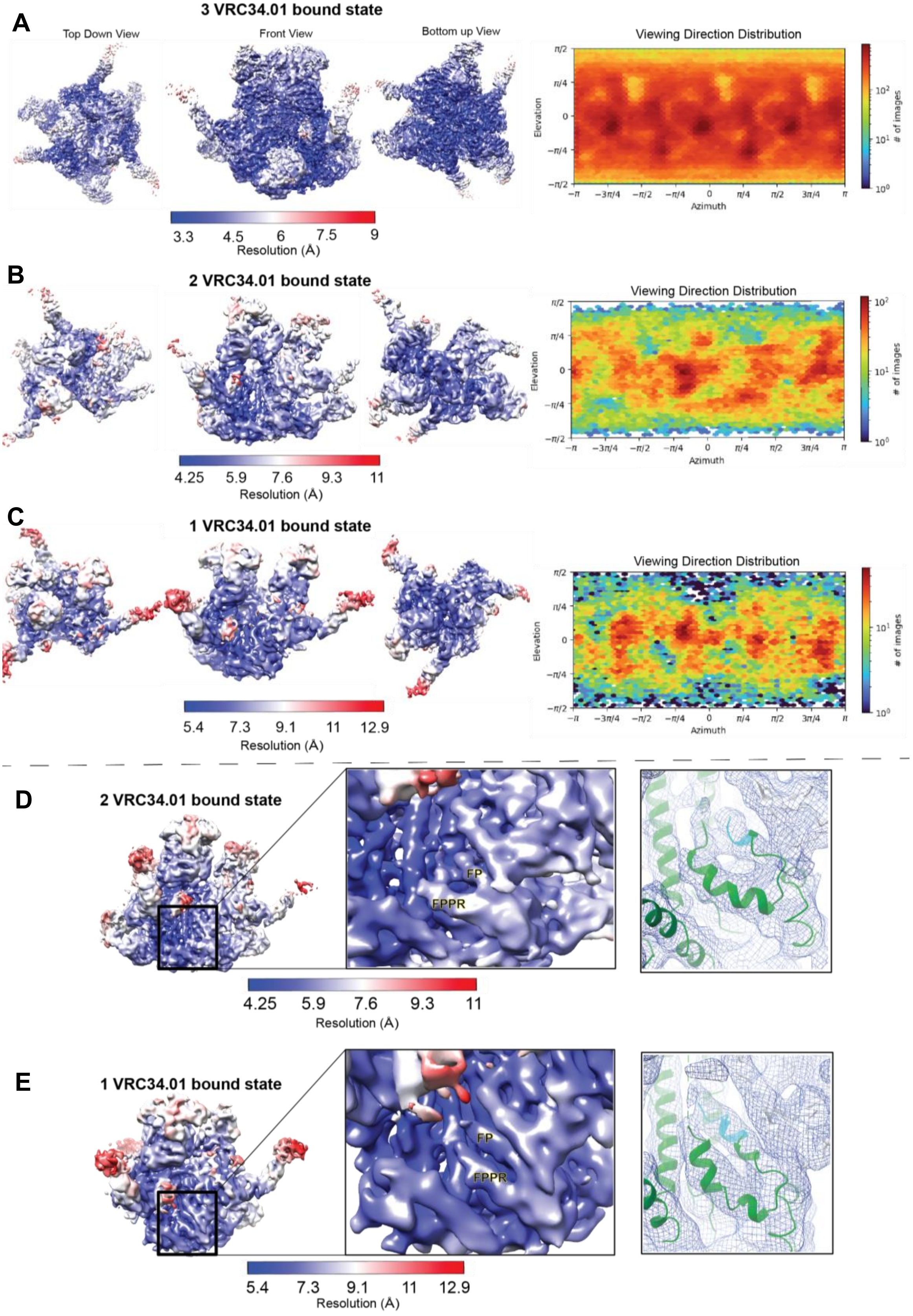
A
3 VRC34.01 bound state
Top Down View
Front View
Bottom up View
Viewing Direction Distribution
Elevation
Azimuth
# of images
3.3 4.5 6 7.5 9
Resolution (Å)
B
2 VRC34.01 bound state
Viewing Direction Distribution
4.25 5.9 7.6 9.3 11
Resolution (Å)
C
1 VRC34.01 bound state
Viewing Direction Distribution
5.4 7.3 9.1 11 12.9
Resolution (Å)
D
2 VRC34.01 bound state
FP
FPPR
4.25 5.9 7.6 9.3 11
Resolution (Å)
E
1 VRC34.01 bound state
FP
FPPR
5.4 7.3 9.1 11 12.9
Resolution (Å)

Supplementary Figure 6. Particle distribution and local resolution estimation for cryo-EM dataset of BG505 SOSIP.664 incubated with CD4 and 17b for 3 days followed by incubation with VRC34.01 Fab for 30 minutes prior to vitrification. **A)** Left. Refined cryo-EM map of Env complex with CD4 and 17b, bound to 3 VRC34.01 Fabs, colored by local resolution. Right. Particle distribution. **B)** Same as in panel A**,** but for the 2 VRC34.01-bound population. **C)** Same as in panel A**,** but for the 1 VRC34.01-bound population. **D** and **E)** Zoomed in view of region around the FP and FPPR in the protomer that was not bound to VRC34.01 in **(D)** the 2-VRC34.01 bound structure (cryo-EM map contoured at a level of 0.111 in ChimeraX), and **(E)** one of the two protomers in the 1-VRC34.01 bound structure that was not bound to VRC34.01 (cryo-EM map contoured at a level of 0.113 in ChimeraX). Zoomed-in panels on the left shown the cryo-EM map colored by local resolution. Panels on the right show the cryo-EM map as a blue mesh with underlying fitted model in cartoon representation with fusion peptide colored cyan. All the structures shown are bound to 3 molecules of CD4 and 3 molecules of 17b Fabs.

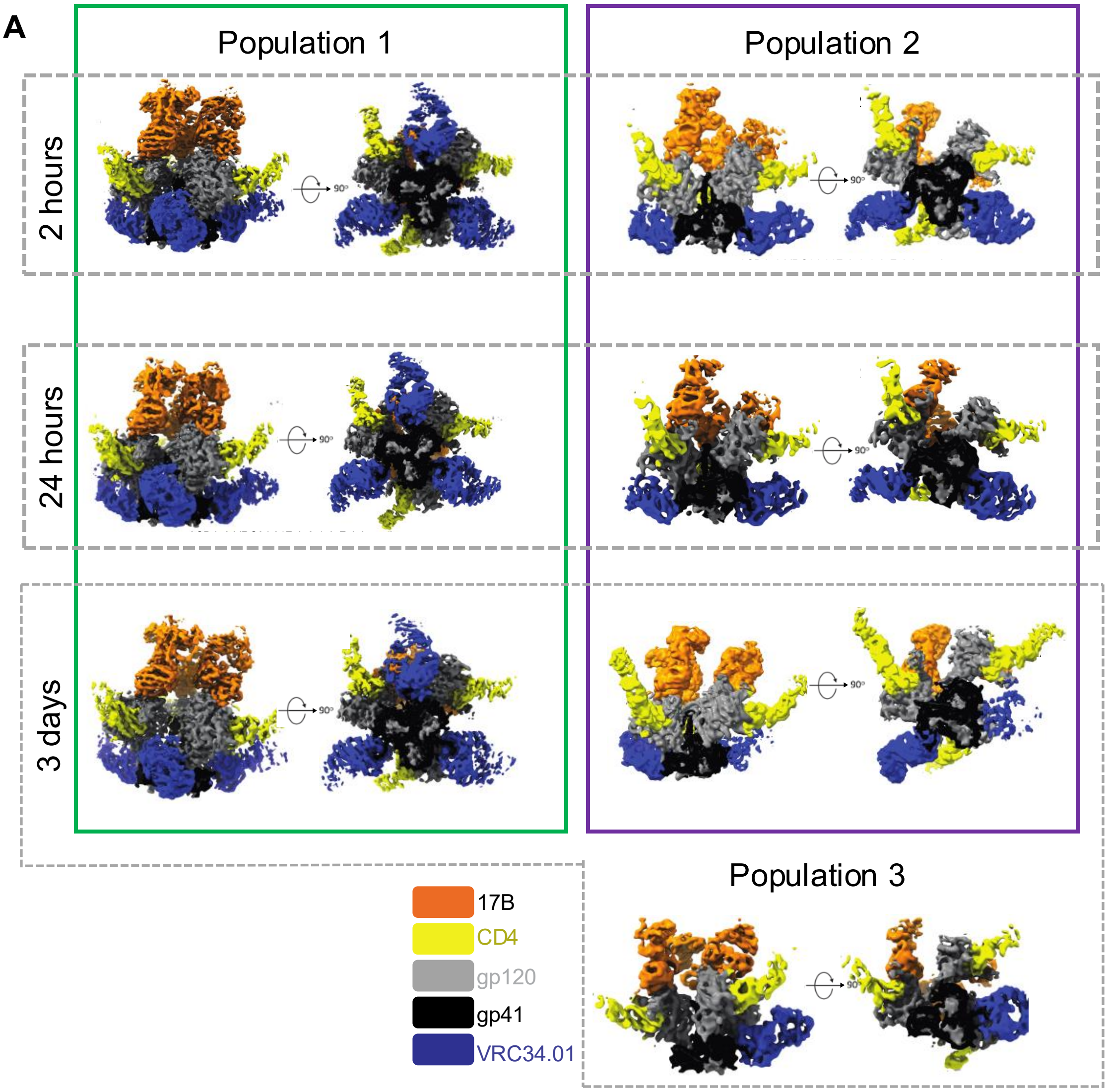

B

	3-CD4, 3-17b Fab, 3-VRC34.01 Fab bound **Population 1**	3-CD4, 3-17b Fab, 2-VRC34.01 Fab bound **Population 2**	3-CD4, 3-17b Fab, 2-VRC34.01 Fab bound **Population 3**
1.3 hours	1,028,496 (94.3 %)	62,272 (5.7 %)	ND
20 hours	335,315 (87.5 %)	47,685 (12.5 %)	ND
3 days	743,776 (89.86 %)	58,240 (7.0 %)	25,613 (3.0 %)

Supplementary Figure 7. Diversity of HIV-1 Env intermediate states visualized by cryo-EM. A) 3D reconstructions of particle populations identified in cryo-EM datasets at different time-points post CD4, 17b incubation of HIV-1 Env followed by addition of VRC34.01 Fab 30 minutes prior to sample vitrification. **B)** Summary of approximate particle numbers and relative percentages of each population identified at each timepoint.

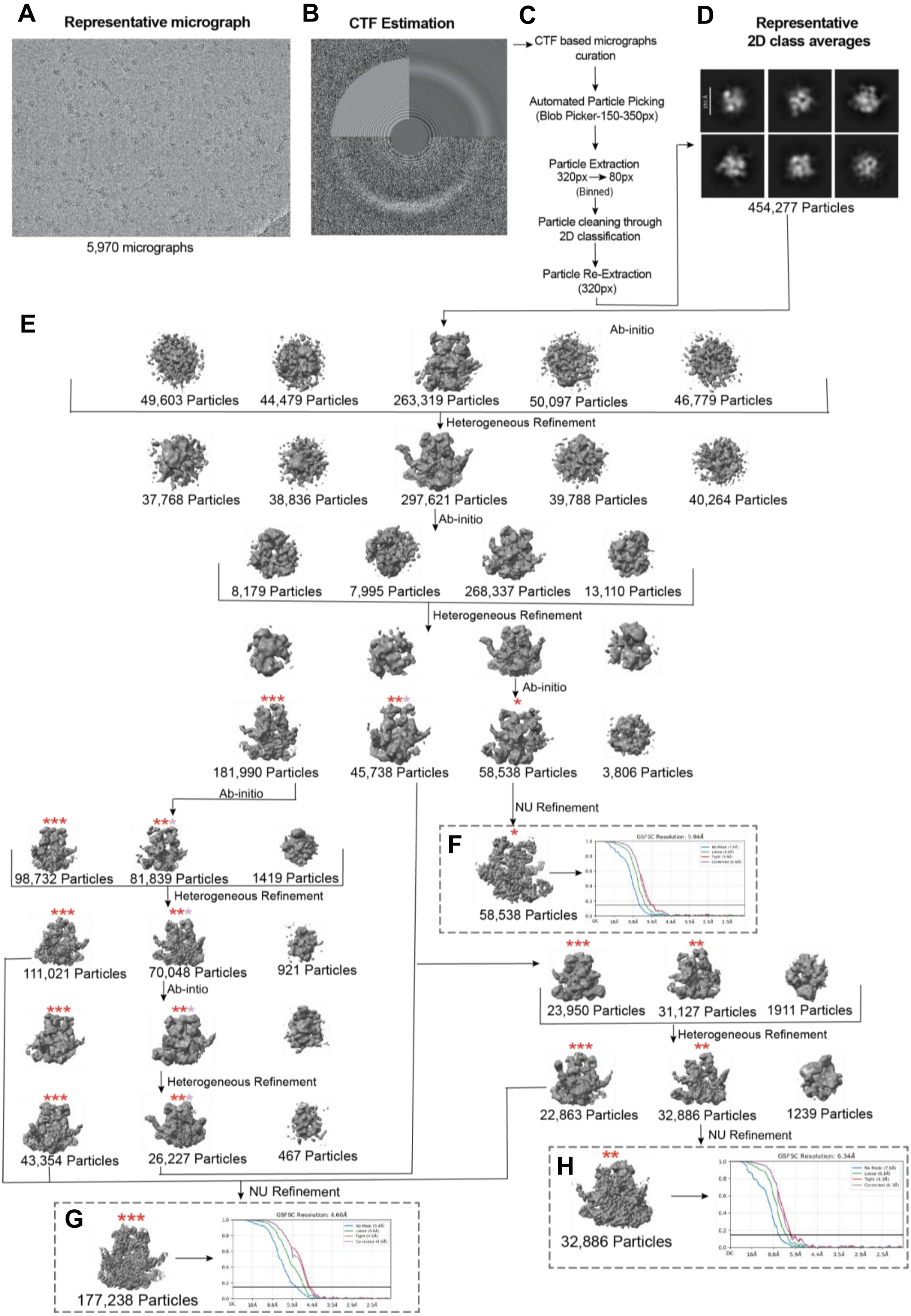
A
Representative micrograph
5,970 micrographs
B
CTF Estimation
C
CTF based micrographs curation
Automated Particle Picking (Blob Picker-150-350px)
Particle Extraction 320px → 80px (Binned)
Particle cleaning through 2D classification
Particle Re-Extraction (320px)
D
Representative 2D class averages
454,277 Particles
E
Ab-initio
49,603 Particles
44,479 Particles
263,319 Particles
50,097 Particles
46,779 Particles
Heterogeneous Refinement
37,768 Particles
38,836 Particles
297,621 Particles
39,788 Particles
40,264 Particles
Ab-initio
8,179 Particles
7,995 Particles
268,337 Particles
13,110 Particles
Heterogeneous Refinement
Ab-initio
181,990 Particles
45,738 Particles
58,538 Particles
3,806 Particles
Ab-initio
NU Refinement
98,732 Particles
81,839 Particles
1419 Particles
Heterogeneous Refinement
F
58,538 Particles
111,021 Particles
70,048 Particles
921 Particles
Ab-intio
23,950 Particles
31,127 Particles
1911 Particles
Heterogeneous Refinement
Heterogeneous Refinement
22,863 Particles
32,886 Particles
1239 Particles
43,354 Particles
26,227 Particles
467 Particles
NU Refinement
NU Refinement
H
32,886 Particles
G
177,238 Particles

Supplementary Figure 8. Cryo-EM data processing workflow for BG505 SOSIP.664 incubated with CD4 and 17b for 2 hours at 37 °C followed by incubation with VRC34.01 Fab for 30 minutes prior to vitrification. **A)** Representative frame aligned micrograph. **B)** Representative contrast transfer function (CTF) fit. **C)** Data Processing workflow, leading to **(D)** Representative 2D class averages from 2D classification, followed by **(E)** 3D classifications and refinements. **F)** Refined map of Env bound to 3 molecules of CD4, 3 molecules of 17b Fabs, and 1 molecule of VRC34.01 Fab (indicated by 1 red asterisks) **G)** Refined map of Env bound to 3 CD4 molecules, 3 molecules of 17b Fab, and 3 molecules of VRC34.01 Fabs. (indicated by 3 red asterisks). **H)** Refined map of Env bound to 3 CD4 molecules, 3 molecules of 17b Fabs, and 2 molecules of VRC34.01 Fabs. (indicated by 2 red asterisks). Fourier shell correlation (FSC) curves for refined 3D maps shown in panels (**F-H)** with the horizontal blue line showing FSC threshold value of 0.143. The red asterisks indicate well-defined density for the VRC34.01 Fab. The pink asterisks indicate partial density for the VRC34.01 Fab.

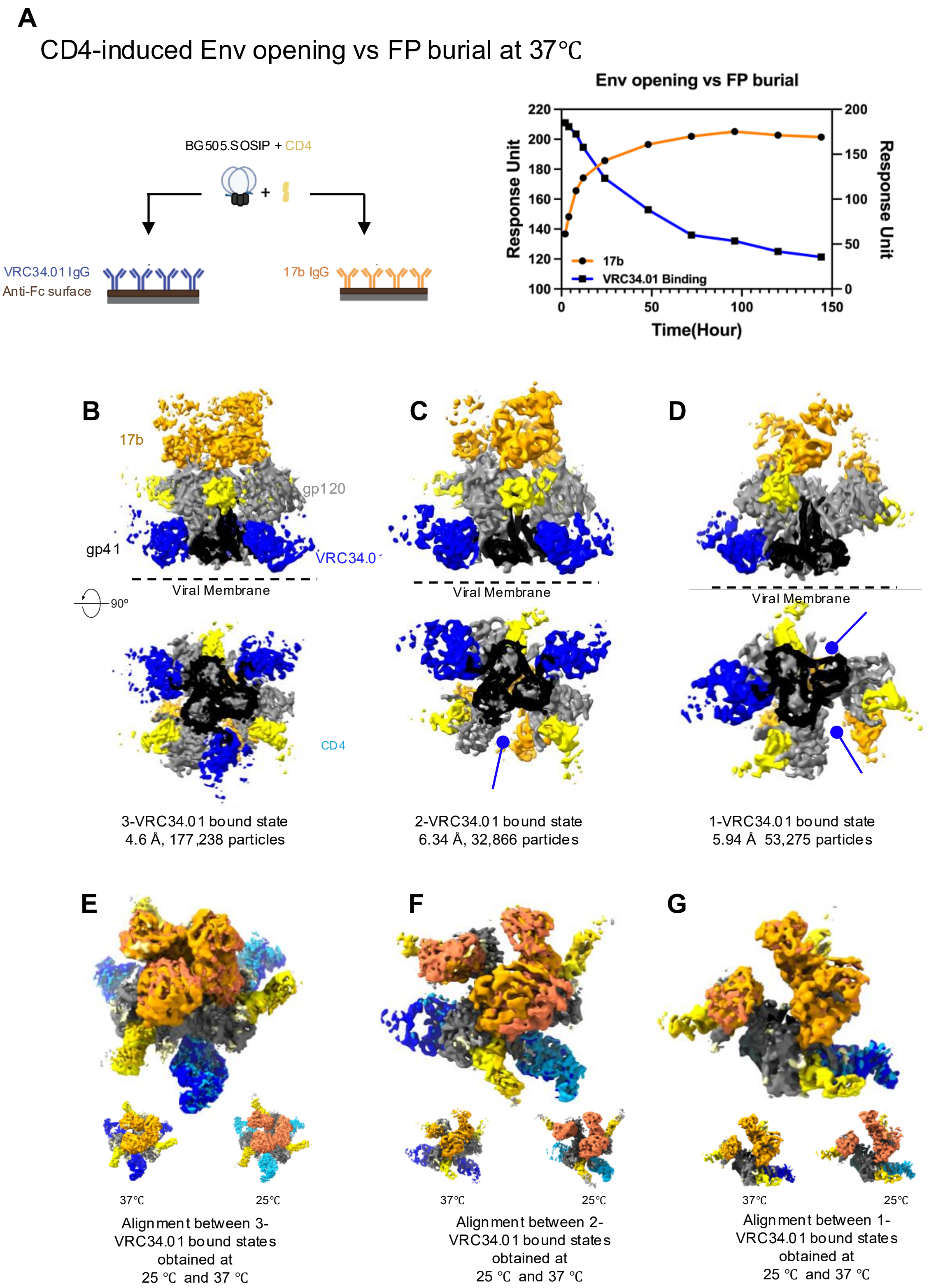
A
CD4-induced Env opening vs FP burial at 37℃
BG505.SOSIP + CD4
VRC34.01 IgG
Anti-Fc surface
17b IgG
Env opening vs FP burial
Response Unit
Response Unit
Time(Hour)
17b
VRC34.01 Binding
B
C
D
17b
gp120
gp41
VRC34.0
Viral Membrane
90°
CD4
3-VRC34.01 bound state
4.6 Å, 177,238 particles
2-VRC34.01 bound state
6.34 Å, 32,866 particles
1-VRC34.01 bound state
5.94 Å 53,275 particles
E
F
G
37℃
25℃
Alignment between 3-VRC34.01 bound states obtained at 25 ℃ and 37 ℃
Alignment between 2-VRC34.01 bound states obtained at 25 ℃ and 37 ℃
Alignment between 1-VRC34.01 bound states obtained at 25 ℃ and 37 ℃

Supplementary Figure 9. Time-dependent conformational changes in HIV-1 BG505 SOSIP Env upon incubation with CD4 at 37 ℃. A) Surface plasmon based binding (SPR) analysis monitoring simultaneous Env opening and fusion peptide burial were measured by incubating Env with CD4 at 37 ℃ and at different time-points injecting over a VRC34.01 IgG or a 17b IgG surface. The SPR measurements were carried out at at 37 ℃. The dataset labeled Repeat 1 in Supplementary Figure 1, panel B was used for plotting the graph. **B-D)** Cryo-EM reconstructions of CD4/17b-bound, partially open Env bound to **B)** 3 VRC34.01 Fabs, **C)** 2 VRC34.01 Fabs and **D)** 1 VRC34.01 Fab. The blue arrows indicate sites unoccupied by VRC34.01. **E)** Alignment of partially open Env states bound to 3-VRC34.01 obtained at 25 ℃ and 37 ℃. **F)** Alignment of partially open Env states bound to 2-VRC34.01 obtained at 25 ℃ and 37 ℃. **G)** Alignment of partially open Env states bound to 1-VRC34.01 obtained at 25 ℃ and 37 ℃. Source data are provided as a Source Data file.

A BG505 SOSIP-PGT122-VRC34.01 (Closed conformation)
PDB: 5I8H

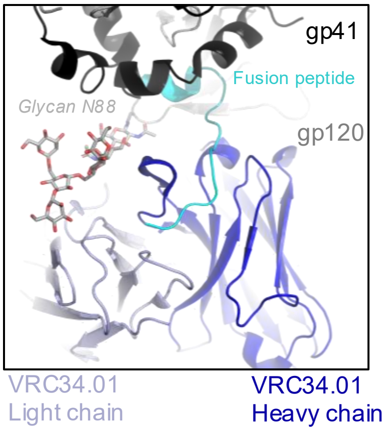

B BG505 SOSIP-CD4-17b-VRC34.01 (Partially open conformation)
PDB: 9D90 (this study)

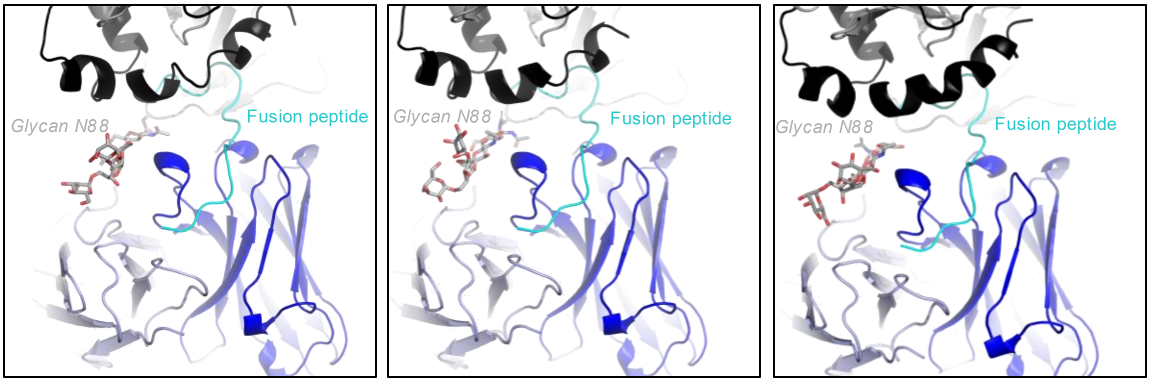

C BG505 SOSIP-CD4-17b-VRC34.01 (Partially open conformation)
PDB: 9D90 (this study)

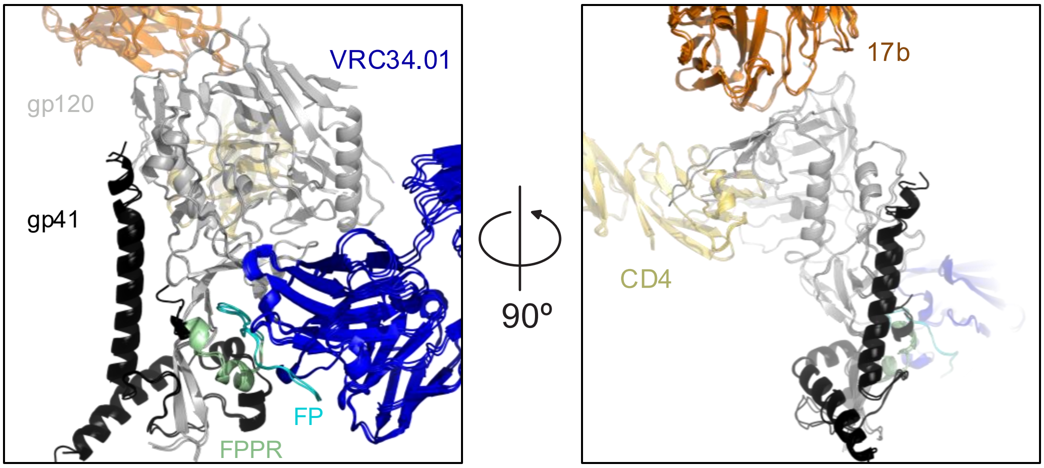

Supplementary Figure 10. Antibody recognition of FP in the context of diverse Env conformations. A) VRC34.01 bound to HIV-1 Env in closed conformation. VRC34.01 heavy and light chains are shown as dark blue and light blue cartoon, respectively; gp120 is colored grey with the glycan at position N88 shown in stock representation; gp41 is colored black; FP s colored cyan. **B)** VRC34.01 bound to HIV-1 Env in partially open conformation. Color scheme is same as in panel **A**. **C)** The three protomers of the Population 1 structure (PDB: 9D90; this study) overlayed by their gp120 subunits. VRC34.01 bound to HIV-1 Env in partially open conformation. Color scheme is same as in panel **A,** except both VRC34.01 heavy and light chains are colored dark blue. FPPR is colored pale green, CD4 yellow and 17b orange.

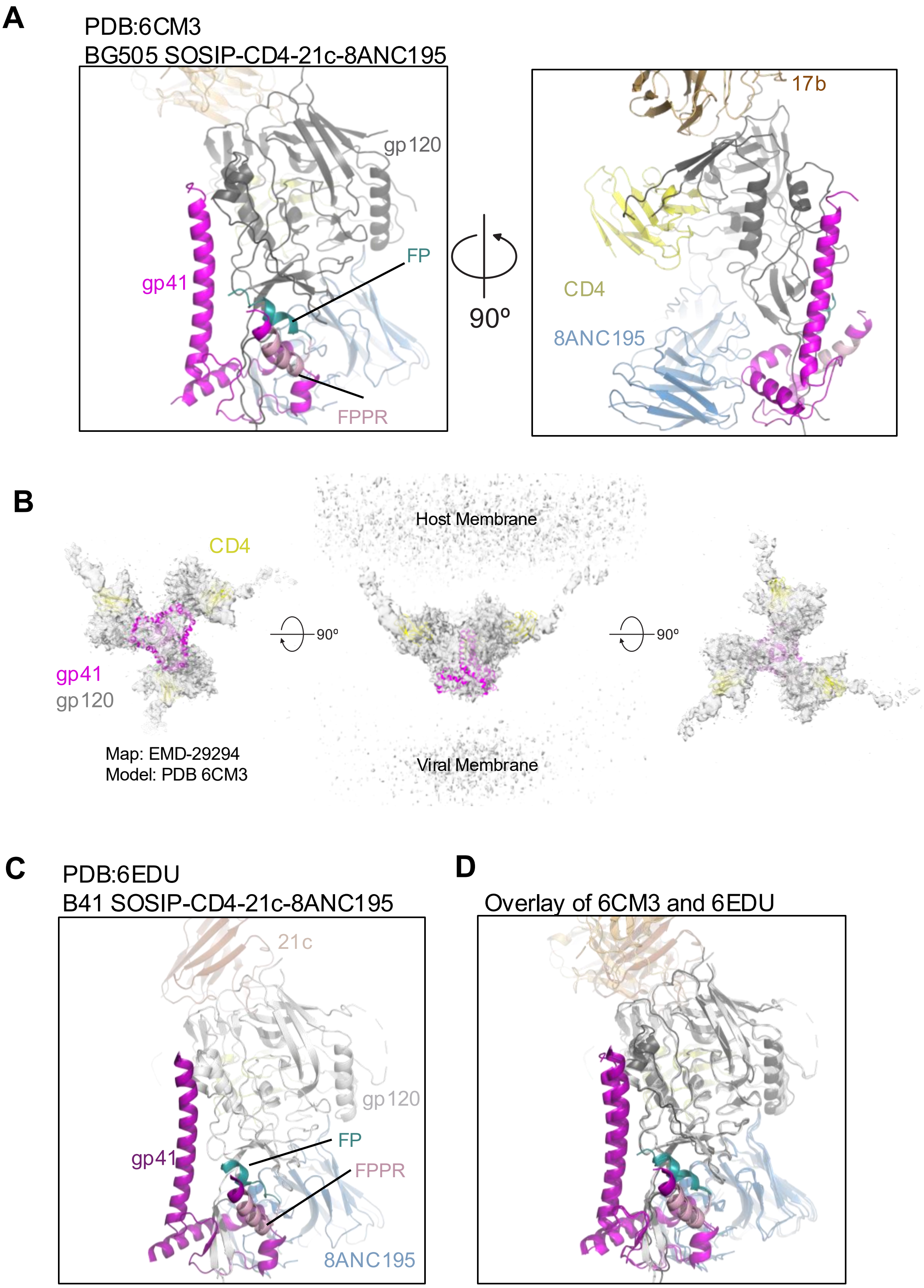
A
PDB:6CM3
BG505 SOSIP-CD4-21c-8ANC195
gp120
FP
gp41
FPPR
90º
17b
CD4
8ANC195
B
Host Membrane
CD4
90º
90º
gp41
gp120
Map: EMD-29294
Model: PDB 6CM3
Viral Membrane
C
PDB:6EDU
B41 SOSIP-CD4-21c-8ANC195
21c
gp120
FP
gp41
FPPR
8ANC195
D
Overlay of 6CM3 and 6EDU

Supplementary Figure 11. Cryo-EM structure of BG505 SOSIP Env bound to CD4, 17b and 8ANC195 (PDB: 6CM3). **A)** One protomer of the partially open Env bound to CD4, 17b Fab and 8ANC195 Fab (PDB ID: 6CM3) shown in cartoon representation zoomed-in at the gp120/gp41 interface. The gp120 subunit is colored dark grey, gp41 magenta, FP dark teal and FPPR light pink. The bound CD4 is colored yellow, 8ANC195 Fab marine blue, 17b Fab brown. **B)** Coordinates of PDB: 6CM3 including Env (gp120 in light gray, gp41 in magenta) and CD4 (yellow) fitted into the *in situ* cryo-ET reconstruction of a partially open CD4-bound Env (EMD-29294). **C)** One protomer of the partially open Env bound to CD4, 21c Fab and 8ANC195 Fab (PDB ID: 6EDU) shown in cartoon representation zoomed-in at the gp120/gp41 interface. The gp120 subunit is colored light grey, gp41 dark purple, FP dark teal and FPPR light pink. The bound CD4 is colored yellow, 8ANC195 Fab marine blue, 21c Fab chocolate. **D)** Structures of BG505 SOSIP bound to CD4, 17b Fab and 8ANC195 Fab, and of B41 SOSIP bound to CD4, 21c Fab and 8ANC195 Fab, overlaid by their gp120 subunits.

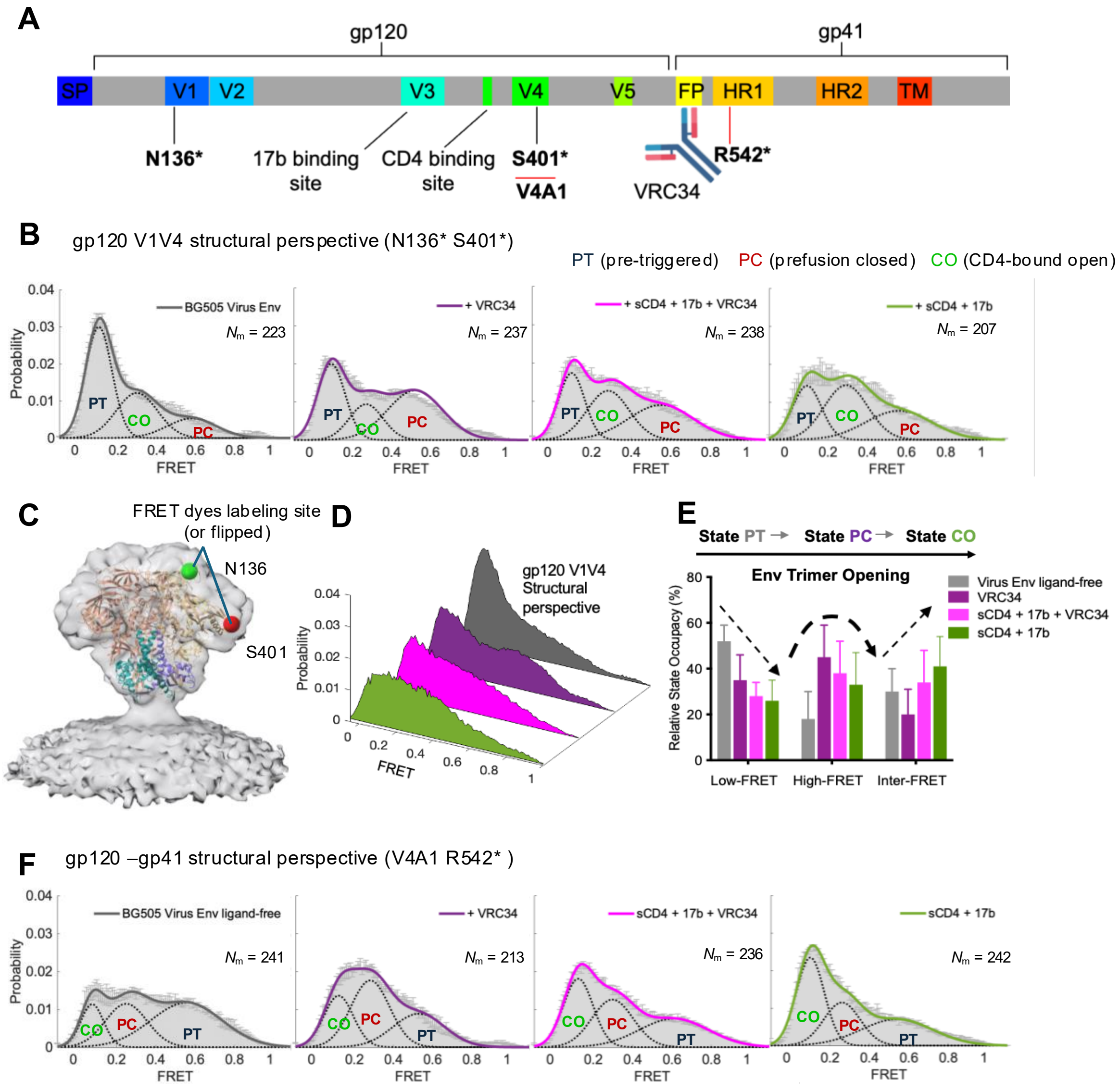

A
gp120
gp41
SP
V1
V2
V3
V4
V5
FP
HR1
HR2
TM
N136*
17b binding site
CD4 binding site
S401*
V4A1
R542*
VRC34
B
gp120 V1V4 structural perspective (N136* S401*)
PT (pre-triggered) PC (prefusion closed) CO (CD4-bound open)
BG505 Virus Env
+ VRC34
+ sCD4 + 17b + VRC34
+ sCD4 + 17b
Nm = 223
Nm = 237
Nm = 238
Nm = 207
Probability
FRET
C
FRET dyes labeling site (or flipped)
N136
S401
D
gp120 V1V4 Structural perspective
Probability
FRET
E
State PT → State PC → State CO
Env Trimer Opening
Virus Env ligand-free
VRC34
sCD4 + 17b + VRC34
sCD4 + 17b
Relative State Occupancy (%)
Low-FRET
High-FRET
Inter-FRET
F
gp120 –gp41 structural perspective (V4A1 R542*)
BG505 Virus Env ligand-free
+ VRC34
sCD4 + 17b + VRC34
sCD4 + 17b
Nm = 241
Nm = 213
Nm = 236
Nm = 242
Probability
FRET

Supplementary Figure 12. Visualizing VRC34-related conformational stabilization and activation of virus Env by smFRET of full-length Env on native virions from two structural perspectives.

A) The domain organization of Env_{BG505} sequence is depicted, with the fluorescent labeling sites used for smFRET and antibody recognition indicated. FRET-paired Cy3/Cy5 fluorophores were site-specifically labeled on V1V4 (N136* S401*) or gp120-gp41 (V4A1 R542*). A1 is a peptide tag, and *indicates an unnatural amino acid incorporation site for click labeling.

B) The conformational distribution-indicated FRET histograms of virus Env_{BG505} probed from gp120 V1V4 (N136* S401*) structural perspectives in the absence or presence of different ligands. Virus Env_{BG505} samples three primary conformational states (PT: Pre-triggered, PC: Prefusion Closed, and CO: CD4-bound open). PT predominates in the ligand-free condition (left panel), while VRC34 (two middle panels) shifts the conformational landscape differently from that of the CD4-bound opening (right panel). FRET histogram represents the mean + s.e.m. determined from three randomly assigned populations of FRET traces (N_m, number of traces).

C - E) Indicated Cy3/Cy5 fluorophore attachment sites in the Env V1-V4 loops (**C**), three-dimensional presentation (**D**) and state occupancy quantifications **(E)** of FRET histograms in B of virus Env_{BG505} observed from the gp120 V1V4 perspective. Relative state occupancies are presented as mean ± s.e.m. determined by estimating the area under each Gaussian curve of histograms in B. For state occupancies and parameters, see Table S4 and Methods.

F) The conformational distribution-indicated FRET histograms of virus Env_{BG505} probed from the gp120 – gp41 (V4A1 R542*) structural perspectives in the absence or presence of different ligands. Virus Env_{BG505} samples three primary conformational states (PT: Pre-triggered, PC: Prefusion Closed, and CO: CD4-bound open). PT predominates in the ligand-free condition (left panel), while VRC34 (two middle panels) shifts the conformational landscape differently from that of the CD4-bound opening (right panel). FRET histogram represents the mean + s.e.m. determined from three randomly assigned populations of FRET traces (N_m, number of traces). Three-dimensional presentation and quantification of FRET histograms observed from the gp120-gp41 perspective are shown in **Figure 4E-G**.

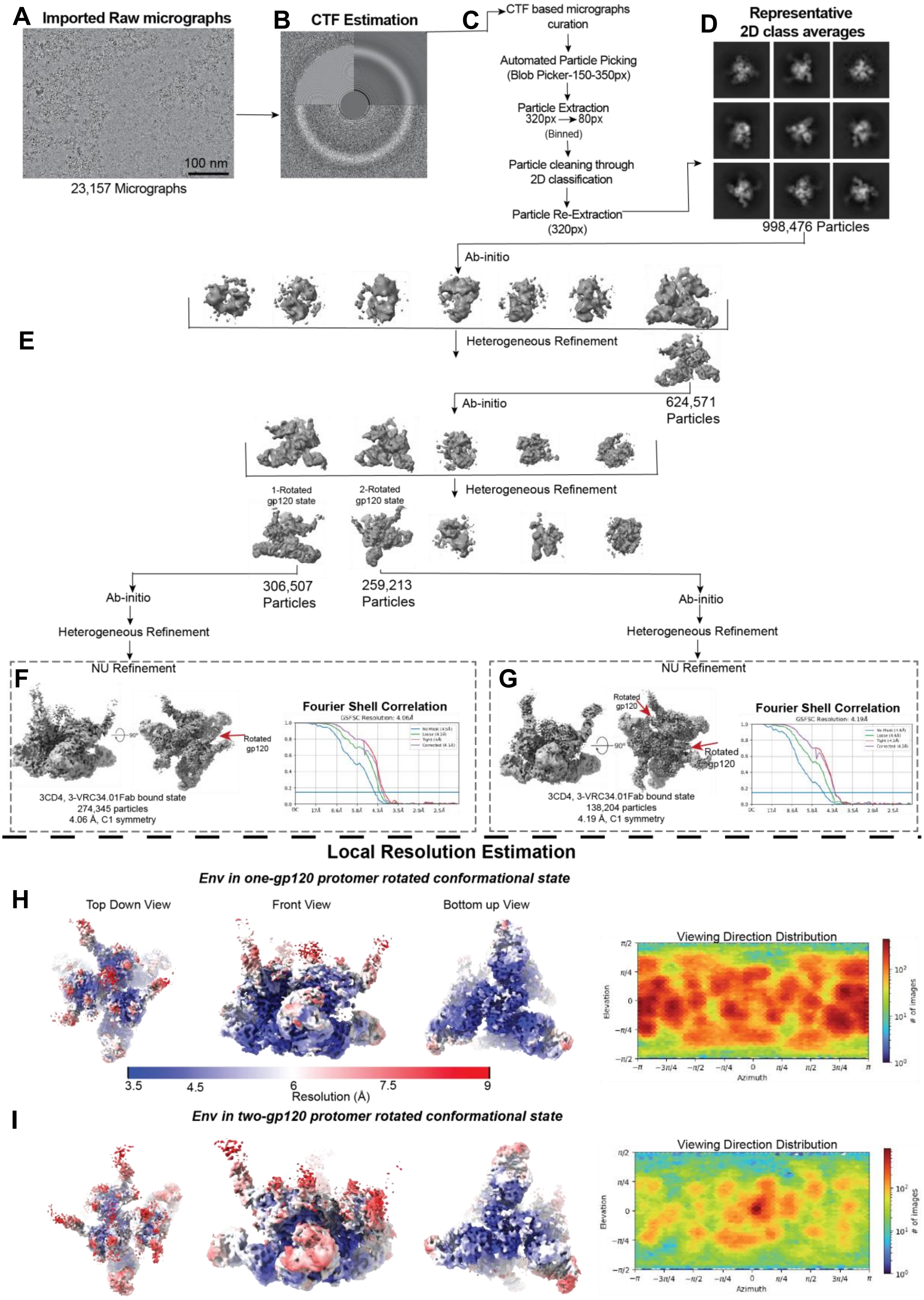
A Imported Raw micrographs
100 nm
23,157 Micrographs
B CTF Estimation
C
CTF based micrographs curation
Automated Particle Picking (Blob Picker-150-350px)
Particle Extraction 320px → 80px (Binned)
Particle cleaning through 2D classification
Particle Re-Extraction (320px)
D Representative 2D class averages
998,476 Particles
Ab-initio
E
Heterogeneous Refinement
Ab-initio
624,571 Particles
Heterogeneous Refinement
1-Rotated gp120 state
2-Rotated gp120 state
306,507 Particles
259,213 Particles
Ab-initio
Heterogeneous Refinement
Ab-initio
Heterogeneous Refinement
F
NU Refinement
Rotated gp120
3CD4, 3-VRC34.01Fab bound state
274,345 particles
4.06 Å, C1 symmetry
Fourier Shell Correlation
G
NU Refinement
Rotated gp120
Rotated gp120
3CD4, 3-VRC34.01Fab bound state
138,204 particles
4.19 Å, C1 symmetry
Fourier Shell Correlation
Local Resolution Estimation
Env in one-gp120 protomer rotated conformational state
H
Top Down View
Front View
Bottom up View
Viewing Direction Distribution
Elevation
Azimuth
# of images
3.5
4.5
6
7.5
9
Resolution (Å)
I
Env in two-gp120 protomer rotated conformational state
Viewing Direction Distribution
Elevation
Azimuth
# of images

Supplementary Figure 13. Cryo-EM data processing workflow for BG505 SOSIP.664 incubated with CD4 for 2 hours at 25 °C followed by incubation with VRC34.01 Fab for 30 minutes prior to vitrification. **A)** Representative frame aligned micrograph. **B)** Representative contrast transfer function (CTF) fit. **C)** Data Processing workflow, leading to, **D)** Representative 2D class averages from 2D classification, followed by, **E)** 3D classifications and refinements. **F)** Refined map of Env bound to 3 CD4 molecules and 3 VRC34.01 Fabs, with one of the three gp120 protomers rotated (indicated with red arrow). **G)** Refined map of Env bound to 3 CD4 molecules and 3 VRC34.01 Fabs, with two of the three gp120 protomers rotated (indicated with red arrows). **H)** Left. Refined map of Env bound to 3 CD4 molecules and 3 VRC34.01 Fabs, with one of the three gp120 protomers rotated, colored by local resolution. Right. Particle distribution. **I)** Left. Refined map of Env bound to 3 CD4 molecules and 3 VRC34.01 Fabs, with two of the three gp120 protomers rotated, colored by local resolution. Right. Particle distribution.
